# The Differences in the Evolutionary Dynamics of MERS and SARS Coronaviruses

**DOI:** 10.3390/v17081114

**Published:** 2025-08-13

**Authors:** Yushan Ding, Jiameng Liu, Jamal S. M. Sabir, Xinyuan Cui, Xuejuan Shen, Nahid H. Hajrah, Mohamed M. M. Ahmed, Meshaal J. Sabir, Onaizan Godian Al-Zogabi, David M. Irwin, Yongyi Shen

**Affiliations:** 1 State Key Laboratory for Animal Disease Control and Prevention, Center for Emerging and Zoonotic Diseases, College of Veterinary Medicine, South China Agricultural University, Guangzhou 510642, China; 2 Guangzhou Zoo & Guangzhou Wildlife Research Center, Guangzhou 510070, China; 3 Centre of Excellence in Bionanoscience Research, King Abdulaziz University, Jeddah 21589, Saudi Arabia; 4 Genomic and Biotechnology Research Group, Department of Biological Sciences, Faculty of Science, King Abdulaziz University, Jeddah 21589, Saudi Arabia; 5 Nucleic Acids Research Department, Genetic Engineering and Biotechnology Research Institute (GEBRI), City for Scientific Research and Technological Applications, Alexandria City 21934, Egypt; 6 Al-Bukairiah General Hospital, Al Bukayriyah 52725, Saudi Arabia; 7 Department of Laboratory Medicine and Pathobiology, University of Toronto, Toronto, ON M5S 1A8, Canada; 8 Banting and Best Diabetes Centre, University of Toronto, Toronto, ON M5S 1A8, Canada; 9 School of Agriculture and Biology, Shanghai Jiao Tong University, Shanghai 200240, China

**Keywords:** SARS-CoV, MERS-CoV, evolutionary dynamics, DPP4

## Abstract

SARS-CoV and MERS-CoV are two coronaviruses that have received significant attention due to their high pathogenicity and mortality rates in human populations. In this study, we compared their evolutionary dynamics to provide a One Health perspective on their differences in terms of the results of disease control. The phylogenetic network of SARS-CoVs showed that human isolates gathered into a “super-spreader” cluster and were distinct from civet isolates. In contrast, dromedary camel- and human-isolated MERS-CoVs were clustered together. Thus, most clades of MERS-CoV can infect humans, and MERS-CoVs seem to more easily spill over the animal-to-human interface. Additionally, the civet can be easily controlled, while the intermediate host (dromedary camels) of MERS-CoV is an important livestock species, so it is impossible to eliminate all animals. This further leads to difficulties in disease control in MERS. Although MERS-CoVs are endemic to dromedary camels in both the Middle East and Africa, human infections are mainly linked to the Middle East. The nucleotide sequences of the MERS-CoV receptor gen (dipeptidyl peptidase 4 (DPP4)) from 30 Egyptians, 36 Sudanese, and 34 Saudi Arabians showed little difference. These findings suggest that the observed disparities in MERS prevalence between populations in the Middle East and Africa may be more strongly attributed to inadequate disease surveillance and the limited camel-to-human transmission of clade C MERS-CoV in Africa, rather than variations in *DPP4* gene.

## 1. Introduction

Coronaviruses (CoVs), a large family of single-stranded RNA viruses, cause respiratory, hepatic, gastrointestinal, and neurologic diseases of varying severity in a wide range of animal species, including humans [1]. Although some CoVs have been known for decades, the potential threat of these viruses to global health security was not fully realized until the outbreaks of Severe Acute Respiratory Syndrome (SARS) and Middle East Respiratory Syndrome (MERS) [2,3,4,5]. These outbreaks were caused by two highly pathogenic coronaviruses, Severe Acute Respiratory Syndrome Coronavirus (SARS-CoV) and Middle East Respiratory Syndrome Coronavirus (MERS-CoV), respectively, which belong to two different subgenuses: Sarbecovirus and Merbecovirus. In 2017, a novel HKU2-related bat coronavirus, swine acute diarrhea syndrome coronavirus (SADS-CoV), caused the death of 24, 693 piglets [6,7]. Notably, the outbreak of severe acute respiratory syndrome coronavirus 2 (SARS-CoV-2) in Wuhan at the end of 2019 raises further concern about these coronaviruses [8,9].

SARS-CoV emerged in 2002 in China, spreading to 29 countries with >8000 infections and nearly 800 deaths [2]. Its transmission chain involves horseshoe bats (*Rhinolophus* sp.) as reservoirs [10] and palm civets (*Paguma larvata*) as intermediate hosts [11,12]. Critically, SARS was contained by 2005 and has not reemerged.

Ten years after SARS-CoV, MERS-CoV emerged in 2012 [13]. While dromedary camels (*Camelus dromedarius*) constitute the established intermediate host for MERS-CoV, bats are recognized as the ultimate evolutionary reservoir [14,15,16]. The receptor utilization profiles differ significantly between these coronaviruses: SARS-CoV employs angiotensin-converting enzyme 2 (ACE2), whereas MERS-CoV binds dipeptidyl peptidase-4 (DPP4) for cellular entry [17]. Crucially, receptor tropism governs coronavirus adaptation dynamics. Recombination events within MERS-CoV’s ancestral receptor-binding domain facilitated receptor switching, enabling emergent variants to acquire human infectivity [18,19]. Notably, recombination events in MERS-CoV’s receptor-binding domain enabled human adaptation. This adaptation allows the sublineage to attach to both bat and human ACE2, highlighting its potential risk for zoonotic transmission [20,21]. SARS-CoV and MERS-CoV share critical epidemiological features: both utilize intermediate hosts and originate from bat reservoirs. Despite SARS containment, MERS persists as a global public health priority [22,23,24]. While prior studies have separately characterized SARS-CoV and MERS-CoV evolution [19,25], a systematic comparison of their evolutionary trajectories, especially in relation to zoonotic spillover efficiency, host adaptation mechanisms, and epidemiological outcomes, remains limited. To examine differences in the epidemic patterns of these two viruses, in this study, we analyze global genomic datasets to compare evolutionary dynamics between these coronaviruses, aiming to elucidate factors driving their differential transmissibility and controllability.

The MERS-CoV is endemic in dromedary camels in the Middle East and Africa. However, all human infection cases have been reported in the Middle East; cases occurring outside this region have involved travelers from the Middle East [16], whereas no African infections have been reported to date. This raises the question of whether genetic differences between Africans and Arabians contribute to differences in susceptibility. Given that DPP4 functions as the cell surface receptor facilitating MERS-CoV entry into cells [26], and considering that adaptive evolution of MERS-CoV is linked to its capacity for human infection [18,19], this study undertook sequencing and comparative analysis of DPP4 alleles from a cohort comprising 30 Egyptians, 36 Sudanese, and 34 Saudi Arabians.

Our work provides the comparative framework linking viral evolution, host ecology, and human genetics to explain differential epidemic control outcomes. These findings directly guide surveillance strategies for high-risk coronaviruses and underscore the need for camel-focused interventions in MERS-endemic regions.

## 2. Materials and Methods

### 2.1. Source of Virus Genomes and Sequence Treatment

All published genomic sequences for MERS-CoV and MERSr-CoV (total 734), SARS-CoV and SARSr-CoV (total 274) were obtained from GenBank (Table S1). SARS-CoV and SARSr-CoV sequences were separated into human, bat, and carnivore groups, based on the host source of the virus. MERS-CoV and MERSr-CoV strains were similarly separated into human, dromedary camel, and bat groups. The complete *ORF1ab*, *S* (spike), *E* (envelope), *M* (matrix), and *N* (nucleocapsid) gene sequences were extracted from each genome and separately aligned using MAFFT v7.505 [27]. The nucleotide diversity (π) of these genes in each population was calculated using MEGA v 11.0.13 [28].

To detect possible recombination signals in coronavirus genomes, seven different detection methods were used: RDP [29], GENECONV [30], BootScan [31], Chimaera [32], MaxChi [33], SiScan [34], and 3Seq [35], all implemented through the Recombination Detection Program (RDP) software version 4.101 [36]. To minimize the chances of false positives, only recombination events confirmed by four or more independent methods were taken into account. Additionally, statistical significance was assessed using a *p*-value threshold of ≤0.05, which was adjusted for multiple comparisons using Bonferroni correction. We analyzed the selective pressure acting on the protein-coding *S*, *E*, *M*, and *N* genes. Nonsynonymous to synonymous substitution ratios (dN/dS) were calculated using MEGA v 11.0.13 [28]. To quantify levels of selection and population expansion, Tajima’s D and Fu & Li’ D * tests were performed using DnaSP v6.12.03 [37]. Models M8 (beta&*w*) and M8a (beta&*w*_s_ = 1) from PAML 4.9 [38] were applied to identify sites that potentially experienced positive selection, where the null model M8a was compared to model M8, which allows positive selection. When likelihood ratio tests for these models were significant (*p* < 0.01), amino acid residues that showed posterior probabilities (PP) > 95% under a Bayes Empirical Bayes (BEB) analysis [39] were regarded as being under positive selection. Simultaneously, the methodologies of Single-Likelihood Ancestor Counting (SLAC) [40], Fixed Effects Likelihood (FEL) [40], Fast Unconstrained Bayesian AppRoximation (FUBAR) [41], and Mixed Effects Model of Evolution (MEME) [42] were utilized with default settings to analyze positive selection through the DataMonkey online platform (http://www.datamonkey.org accessed on 26 June 2025). The *p*-value of MEME, FEL, and SLAC was 0.1, and that of FUBAR was 0.9. Positive selection, neutral selection and negative selection were defined as dN/dS > 1, dN/dS = 1, and dN/dS < 1 respectively. To reduce the influence of confounding factors on selection analysis, sequences showing possible recombination signals, as detected by the RDP4 software, were removed from consideration. Results that were supported by at least three of the mentioned algorithms across these five methods were considered reliable.

The haplotype network for the *S* gene sequences from MERS-CoV and SARS-CoV genomes was constructed using PopART version 1.7 [43].

### 2.2. DNA Extraction, PCR, and Sequencing the DPP4 Gene

Blood samples from 30 Egyptians, 36 Sudanese, and 34 Saudi Arabians were collected. The study was approved by the research ethics committee of King Abdulaziz University. Genomic DNA was extracted using the standard phenol/chloroform method. To amplify the *DPP4* gene, a total of 14 pairs of primers were designed (Supplementary Table S2). The primers produced a total gene length of 13, 474 base pairs, including all exon regions and 10, 208 base pairs of intronic sequences, accounting for 16.4% of the complete *DPP4* gene, which is 81, 971 base pairs long. PCR was performed in a 50 µL volume containing 25 µL of 2× PrimeSTARTM GC Buffer, 0.25 mM dNTPs, 0.2 µM of each primer, 1.5 U PrimeSTARTM HS DNA Polymerase (TaKaRa Biosystems, Dalian, China), and 100 ng genomic DNA. The PCR amplification profile was 95 °C for 5 min, followed by 35 cycles of 98 °C for 10 s, 53 °C for 15 s, and 72 °C for 1 min, with a final extension for 10 min at 72 °C. PCR products were visualized on 1.0% agarose gels, purified on spin columns (Watson Biotechnologies Inc., Shanghai, China), and directly sequenced for both strands using a BigDyeTM Terminator Cycle Sequence Kit (ABI Applied Biosystems 3730, Foster City, CA, USA) according to the manufacturer’s manual. DNA sequences were edited using DNAstar software (version 11.1, DNASTAR Inc., Madison, WI, USA), with the newly determined sequences deposited into GenBank (accession numbers: MK670823–MK670922).

## 3. Results

### 3.1. The Evolutionary Characteristics of SARS-CoVs

**Figure 1:**
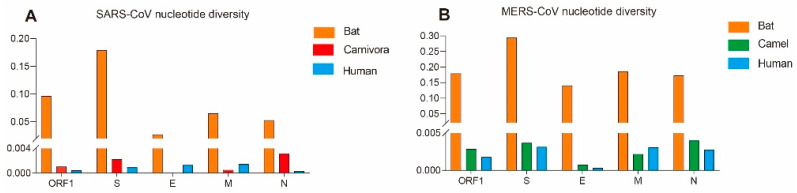
Nucleotide diversity in the *ORF1ab*, *S*, *E*, *M*, and *N* genes of SARS-CoV and MERS-CoV isolated from bats, carnivores (for SARS-CoV) or camels (for MERS-CoV), and human hosts. (**A**) SARS-CoVs. (**B**) MERS-CoVs.

Of the SARS-CoV gene examined, the bat-isolated SARS-CoV lineages exhibit significantly higher nucleotide diversity (π) than human and carnivore isolates (Figure 1A), indicating prolonged stable coexistence within their natural reservoir host with the accumulation of neutral mutations. Human-isolated SARS-CoV demonstrates exceptionally low genetic diversity (π < 0.001), particularly in the S gene (π = 0.00094) (Table 1). This pattern suggests a stringent genetic bottleneck following cross-species transmission, with only limited variants carrying critical adaptive mutations at key sites including S protein positions 479 and 487, initiating super-spreading events. Consequently, enhanced surveillance of bat coronaviruses is warranted to preempt the emergence of human-adapted recombinants.

To test for neutrality in the evolution of SARS-CoV sequences, we performed Tajima’s D and Fu and Li’s D * tests. Significant negative values for Tajima’s D and Fu and Li’s D * indicate deviations from neutrality, suggesting selective sweeps and/or population expansions. Tajima’s D and Fu and Li’s D * values calculated from *ORF1*, *S*, *M*, and *N* genes isolated from humans were significantly less than zero. The dN/dS ratios for *S* genes from carnivores and *M* genes from humans exceeded 1. Site model tests revealed that the *S* genes of bats, carnivores, and humans have undergone positive selection, with a series of positively selected amino acid sites identified. Notably, bat SARS-CoV lineages exhibited signatures of adaptive evolution at residues 8, 22, 25, 81, 268, 410, and 540 of the N protein (Table 2).

**Figure 2:**
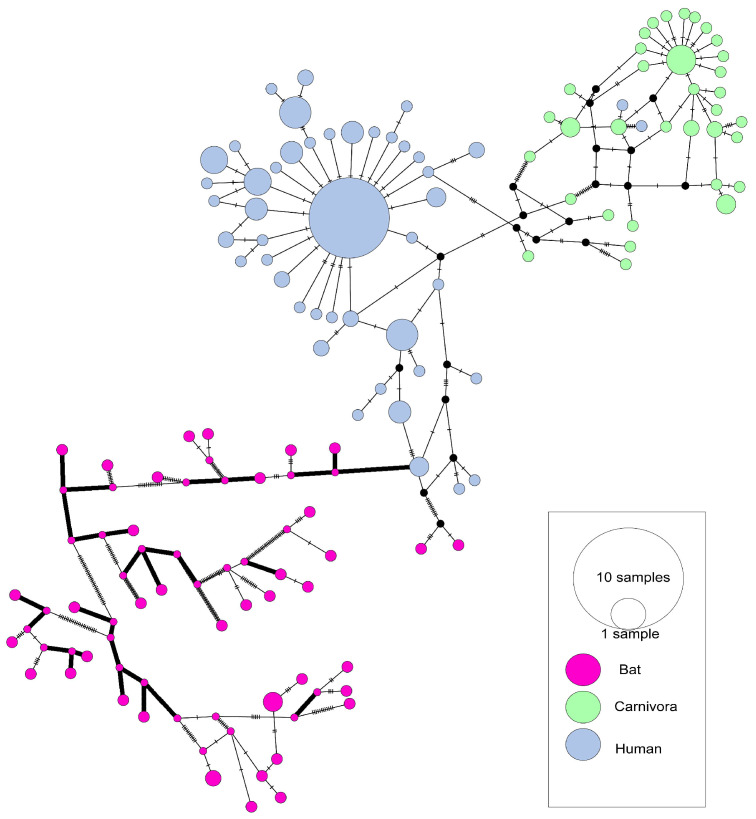
Haplotype network of the *S* gene from SARS-CoV and SARSr-CoV genomes. (Host).

The haplotype network based on the *S* gene of SARS-CoVs showed that the human-isolated SARS-CoV sequences were mainly isolated in 2003 and were particularly concentrated (Figure 2). In total, 47 of 144 human SARS-CoV sequences in the analysis were related to the central haplotype (the biggest blue cycle in Figure 2). Those strains that cause super transmission (HZS2-C, HZS2-D, Sin2500, Sin2677, Sin2748, CUHK-Su10, CUHK-AG01, CUHK-AG03, CUHK-AG03) were all in this haplotype. Therefore, most human infection cases arose due to this cluster. In contrast, bat isolates exhibited substantially higher genetic divergence (π = 0.17954 vs. 0.00094) (Table 1), while carnivore sequences formed a discrete clade proximal to human variants. The time-scaled haplotype network analyses of SARS-CoV revealed a super transmission among 2003 human infection haplotypes (Supplemental Figure S1), indicating superspreader events. The 2004 human haplotypes nested within 2003 strains, confirming sporadic laboratory-origin infections. Persistent intermingling of 2003–2004 animal haplotypes demonstrated continuous viral evolution within reservoir hosts [25].

### 3.2. The Evolutionary Characteristics of MERS-CoV

Consistent with SARS-CoV evolutionary patterns, MERS-CoV genes from human and dromedary camel hosts exhibit significantly lower nucleotide diversity than bat-derived variants (Figure 1B), reaffirming that bats are genetically diverse reservoirs. While most genes show comparable diversity between human and camel isolates, the envelope (*E*) gene displays reduced diversity in human strains. Given its short length (249 bp), *E* gene diversity metrics may be disproportionately sensitive to minor sequence variations, potentially limiting the biological significance of this observed difference. Crucially, key structural genes (*S*, *M*, *N*) maintain similar diversity in both hosts (π ≈ 0.003), indicating sustained bidirectional transmission without major genetic bottlenecks. Values for Fu and Li’s D * calculated for all genes from human and dromedary camel isolates were significantly less than zero. The dN/dS value for the *E* gene from dromedary camel was greater than 1 (Table 1). Camel-derived variants exhibited adaptive changes at positions 26, 28, 424, 459, 723, and 1224 of the S protein. Furthermore, camel MERS-CoV lineages showed evidence of positive selection at position 69 in the M protein and residue 3, 198 in the N protein. Bat-associated strains displayed positive selection adaptations at residues 200, 328, 389, and 424 of the N protein, whereas no positive selection sites were identified in human strains (Table 3).

**Figure 3:**
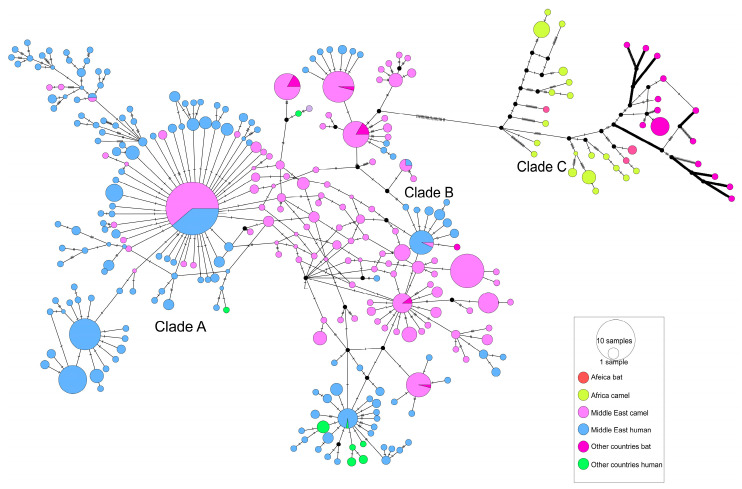
Haplotype network of the *S* gene from MERS-CoV and MERSr-CoV genomes. (Host).

A haplotype network was constructed using MERS-CoV S gene sequences. A total of 308 haplotypes were identified in these sequences. As shown in Figure 3, Middle Eastern dromedary camel MERS-CoVs were divided into multiple clusters, most of which were shared between dromedary camels and humans. African dromedary camel MERS-CoVs formed a distinct single cluster (clade C) that differs from MERS-CoVs present in humans and camels from other regions (clades A and B). In the time-scaled haplotype network, analyses of MERS-CoV showed a 2014–2015 haplotype surge (Supplemental Figure S2), correlating epidemiologically with the 2014 Saudi Arabian outbreak and 2015 Korean nosocomial transmission [19,44]. Subsequent endemicity was evidenced by temporally diffuse haplotypes without chronological gradients, suggesting frequent zoonotic reintroductions and sustained local transmission with recurrent viral gene flow.

### 3.3. DPP4 Did Not Show Any Difference Between Arabs and Africans

**Figure 4:**
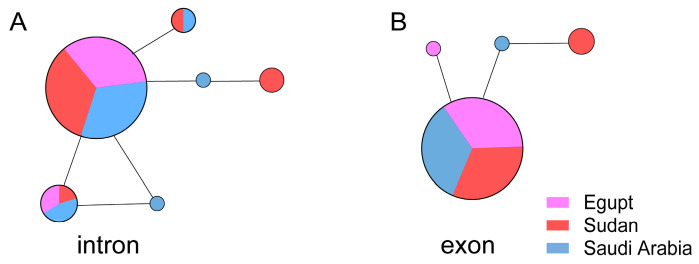
Haplotype networks of exon and intron regions of *DPP4* gene. (**A**) Intron region. (**B**) Exons.

DPP4 exon and partial intron sequences, in 14 fragments, were obtained from 30 Egyptian, 36 Sudanese, and 34 Saudi Arabian specimens and were sequenced. We identified four exon haplotypes and six intron haplotypes in these sequences. The central haplotype for both the exon and intron sequences was shared by the Egyptians, Sudanese, and Saudi Arabians (Figure 4). The genetic diversity of the exons and introns was 0.0~0.3% and 0.0~0.1%, respectively, indicating that there was a very limited difference in the DPP4 sequences between Arabs and Africans.

## 4. Discussion

Bats are the reservoir hosts for both SARS-CoV and MERS-CoV, which then use palm civets and dromedary camels as intermediary hosts before dissemination to humans, respectively [45,46]. Although they both have the same reservoir host and use intermediary hosts before dissemination to humans, SARS was controlled quickly while MERS continues to have sporadic human infections.

The genetic diversity of all genes from human- and carnivore-isolated SARS-CoVs was significantly lower than that of bat-isolated SARS-CoVs (Figure 1A). Only a subset of these viruses appears capable of transmission through carnivores and subsequent infection of humans. This suggests that most SARS-CoVs are likely highly incompatible with humans and palm civets. Tajima’s D and Fu and Li’s D * values indicated population expansion in human isolates, consistent with the rapid spread of human infections.

In the SARS-CoVs haplotype network diagram, bat, carnivore, and human isolates form distinct clusters, each representing an independent evolutionary branch. Bat-isolated SARS-CoVs exhibit significant genetic diversity, with multiple haplotypes separated by extended branches (Figure 2), indicating high population diversity and prolonged evolutionary history within their natural hosts. The carnivore and human clusters are directly connected by short branches (≤3 mutation steps), supporting the role of civets as intermediate hosts. Concurrently, we identified multiple sites under positive selection pressure, including residues 147, 227, 462, 479, and 609 within the S protein of isolates SARS-CoV derived from carnivorous hosts (Table 2). This indicates that SARS-CoV underwent adaptive evolution in carnivore hosts prior to zoonotic spillover into humans [31]. Human-isolated SARS-CoVs haplotype form an independent, star-shaped radiation cluster, indicating the presence of super-spreaders, consistent with epidemiological evidence [47]. The cross-species transmission of SARS-CoV is dependent on adaptive evolution within carnivore hosts, with human infections predominantly arising from a single dominant viral lineage. Thus, controlling intermediate hosts may effectively interrupt viral transmission. Network topology analysis indicates a limited potential for direct zoonotic transmission from bats to humans; nevertheless, intensified surveillance at wildlife–animal market interfaces remains essential.

Unlike SARS-CoVs, the MERS-CoVs haplotype network comprises three distinct evolutionary clades: clade A, clade B, and clade C. In clades A and B, Middle Eastern camel and human-isolated MERS-CoVs haplotypes are extensively intermingled and shared (Figure 3), with camel- and human-isolated haplotypes connected by short branches (≤3 mutation steps), supporting sustained camel-to-human transmission in the Middle East, consistent with epidemiological observations [14,48]. African camel-isolated MERS-CoVs haplotypes cluster predominantly within clade C and are connected to Middle Eastern camel-isolated haplotypes via distinct long branches, indicating geographical isolation. Additionally, clade C exhibits a markedly low prevalence among African populations. Previous studies have demonstrated that recombinant MERS-CoV carrying the spike (S) protein from the Ethiopian isolate exhibits a reduced virulence phenotype, characterized by enhanced neutralization sensitivity and reduced replication kinetics, compared to variants expressing the S protein from the Middle Eastern EMC strain [49]. Crucially, a single amino acid substitution in the RBD reversed the neutralization profile, indicating that minor genetic variations may substantially influence MERS-CoV adaptability. These findings suggest that reduced human-to-human transmission of clade C strains may result from adaptive constraints driven by antigenic drift [50]. However, limited surveillance capacities in African regions endemic for clade C (e.g., Sudan and Kenya), where diagnostic capacities fall below WHO standards, may result in the underreporting of mild or asymptomatic infections—a key confounding factor in transmission assessment [51].

Compared with SARS, MERS has been a much longer epidemic. Specific antibodies against MERS-CoV have been detected in the serum of dromedary camels from Africa and the Arabian Peninsula collected since 1992 [52,53], suggesting that MERS-CoV has had a long co-evolutionary time to develop a bat–dromedary camel–human transmission route. Comparable genetic diversity in human- and camel-isolated MERS-CoVs (Figure 1B) and shared clades demonstrates multi-lineage spillover capability (Figure 3), a conclusion supported by previous studies [14]. This viral multi-lineage spillover capability enhances zoonotic risk, constituting the fundamental barrier to MERS eradication. Moreover, from a socioeconomic perspective, the carnivore-intermediate amplifying host of SARS-CoV was easy to control by closing wild animal markets; meanwhile, the dromedary camel, as an important livestock species in Arab countries, plays key roles in transportation, food, fabric (wool), and entertainment, meaning that it could not be so easily controlled [54]. It is likely impossible to eliminate all dromedary camels from Arab countries. Serological surveys have also showed that there is a high prevalence of MERS-CoV-neutralizing antibodies in dromedary camels [55,56]; therefore, unlike SARS-CoV, the spread of MERS-CoV has a stable natural reservoir, meaning that its epidemic may last longer. Eradication of MERS-CoVs from the dromedary camels is the primary condition needed for the control of this disease in the Arabian Peninsula. Mitigation of MERS-CoV transmission necessitates integrated One Health interventions, with three evidence-based strategies emerging as critical within a global surveillance framework. (1) Accelerating camel vaccine development (e.g., S protein-based subunit vaccines) and implementing vaccination programs, prioritizing camels in slaughterhouses, markets, and high-density husbandry systems to reduce zoonotic spillover [14,54]. (2) Enhancing camel trade biosecurity through mandatory MERS-CoV nucleic acid testing (e.g., real-time RT-PCR) for imported camels, the isolation of positive cases, and the temporary suspension of the cross-regional live camel trade during outbreaks [57]. (3) Implementing risk-stratified camel–human contact management, including mandatory personal protective equipment (PPE) (N 95 masks, goggles) for camel handlers and slaughterhouse workers, regular screening, and sentinel surveillance in camel-rearing regions to address surveillance gaps [58].

To investigate the genetic basis of host jumps, a series of adaptive evolutionary analyses were performed. The *S* gene of SARS-CoVs from bat, carnivore, and human isolates, as well as the *S* gene of MERS-CoVs from dromedary camels, showed evidence of positive selection (Table 2 and Table 3). Although SARS-CoV and MERS-CoV utilize distinct receptors [59,60,61], their S proteins form spikes on the surface of coronavirus particles that mediate receptor recognition and are critical for viral entry into host cells, thereby altering viral tropism [2,62]. Antigenic drift and genetic recombination of the S protein should help viruses recognize receptors in the new hosts, a conclusion supported by previous studies [12,19,63]. SARS-CoV and MERS-CoV exhibit host-adapted spillover mediated by intermediate hosts [25,64], whereas SARS-CoV-2 demonstrates accelerated adaptive evolution facilitated by global transmission networks, reflecting distinct epidemiological trajectories. Given that SARS-CoV-2 is unlikely to be the last coronavirus to cross species barriers, we emphasize that future pandemics may originate from recombination events in under-monitored animal reservoirs. Consequently, sustained vigilance at zoonotic interfaces is imperative [65].

Although MERS-CoV is endemic to dromedaries in Africa [66,67,68,69,70,71,72], human MERS-CoV infections in African countries—including East Africa (Kenya, Sudan), West Africa (Ghana, Nigeria), and North Africa (Morocco, Tunisia, Egypt)—are very rare [73]. A key determinant of viral species tropism is the host cell entry level, which is mediated by the binding of the MERS-CoV spike protein to DPP4 on host cells [60,61]. We therefore compared DPP4 allele sequences from Arabs and North Africans to determine whether differences in DPP4 sequences might explain variations in susceptibility. However, no differences in DPP4 alleles were observed between these two populations (Figure 4). The reported difference in susceptibility between individuals from the Middle East and Africa may instead be attributed to inadequate disease surveillance in Africa [73,74], or the low camel-to-human transmission of clade C MERS-CoV in Africa [73], rather than differences in the DPP4 gene. In addition to the diversity of *DPP4* genes, cytokine-related genes (such as *TNF-α* and *IL-6*), HLA (human leukocyte antigen), and innate immune receptor genes may also regulate susceptibility to coronaviruses [75,76,77]. For instance, the alleles HLA-DQA1*01:03 and DQB1*06:01 are commonly found in Asian populations, particularly in southern China, and are linked to a higher risk of severe SARS-CoV-2 infection [78,79]. HLA-DRB111:01 and DQB102:02 have been identified in the Saudi Arabian population and are linked to mild cases of MERS [80]. Future studies should investigate other genetic factors to explore their possible connections to differences in MERS infection rates across populations in the Middle East and Africa.

In summary, our study has compared the evolutionary characteristics of SARS-CoV and MERS-CoV. Most SARS-CoVs are incompatible with humans, and most human SARS-CoV infections are mainly caused by “super-spreaders.” The intermediate host (civets) in the SARS-CoV epidemic is an exotic animal, which is easy to control to reduce new human infections. In contrast, all major MERS-CoV clades demonstrate zoonotic competence. Critically, MERS-CoV’s intermediate host—dromedary camels—constitutes an economically vital livestock species in Arab nations. High seroprevalence in camel populations (>70% in endemic regions) sustains persistent spillover risk. These combined factors impede MERS containment. Notably, DPP4 protein sequences exhibit no differences between Arabs and Africans; thus, there is no genetic evidence supporting the notion that Africans are less susceptible to MERS-CoV infection. These findings indicate that African countries should enhance MERS-CoV surveillance by expanding its scope—particularly among individuals with frequent contact with dromedary camels—to determine the true extent of MERS-CoV infections in Africa.

## Figures and Tables

**Table 1:** Genetic diversity and neutrality analyses of the S, E, M, and N genes from SARS-CoV and MERS-CoV.

	Gene	Host	Nucleotide Diversity (π)	Neutrality Analyses	
				Fu and Li’s D *	Fu and Li’s D *
SARS-CoV	*ORF1*	Human	0.00048	−2.63679 **	−5.05802 **
		Bat	0.09634	−0.72289	−0.85435
		Carnivora	0.00107	−1.45552	−1.05296
	*S*	Human	0.00094	−2.29683 **	−4.31580 **
		Bat	0.17954	0.35301	−0.36182
		Carnivora	0.00225	−0.10083	−1.05012
	*E*	Human	0.00132	−1.63734 *	−0.82508
		Bat	0.02742	−1.05697	−1.11301
		Carnivora	/	/	/
	*M*	Human	0.00151	−1.99699 *	−3.68341 **
		Bat	0.06514	−1.22163	−1.01881
		Carnivora	0.00054	−1.03789	−0.50381
	*N*	Human	0.00037	−2.23097 **	−4.79424 **
		Bat	0.05313	−1.43029	−1.77074 **
		Carnivora	0.00320	−0.63115	0.04240
MERS-CoV	*ORF1*	Human	0.00180	−2.32084 **	−8.53056 **
		Camel	0.00286	−2.03977 *	−6.02846 **
		Bat	0.17953	−0.04466	0.68164
	*S*	Human	0.00316	−2.23642 **	−7.86620 **
		Camel	0.00369	−2.12122 **	−5.43913 **
		Bat	0.29554	−0.13075	0.44654
	*E*	Human	0.00033	−2.06853 *	−5.60248 **
		Camel	0.00078	−1.89892 *	−3.04304 *
		Bat	0.14117	0.35028	0.68165
	*M*	Human	0.00307	−1.65090	−5.28654 **
		Camel	0.00219	−2.08234 *	−3.89494 **
		Bat	0.18639	0.06353	0.68401
	*N*	Human	0.00276	−2.15013 **	−4.22054 **
		Camel	0.00401	−2.10318 *	−4.26757 **
		Bat	0.17372	−0.07776	0.54725

Note: * p ≤ 0.05; ** 0.05 > *p* > 0.001.

**Table 2:** Selective pressure analyses of the S, E, M, and N genes from SARS-CoV.

Gene	Positive Selection Pressure Sites Identified by Different Methods					
	M8 vs. M8a	FEL	SLAC	FUBAR	MEME	PSC *
Bat S	5, 8, 20, 132, 133, 135, 136, 138, 154, 182, 195, 411, 412, 422, 439, 481, 503, 589, 689	166, 540, 596, 633	None	451, 540, 648	7, 20, 24, 43, 81, 82, 83, 89, 91, 101, 130, 156, 157, 160, 166, 174, 178, 185, 190, 207, 210, 231, 242, 252, 314, 410, 411, 451, 454, 464, 465, 477, 501, 502, 509, 516, 540, 562, 596, 633, 648, 689, 693, 753, 876, 935, 1117, 1250, 1253	**540**
Bat E	None	None	None	None	None	-
Bat M	14	97	None	None	97	-
Bat N	8, 22, 268, 410	8, 22, 25, 34, 81, 121, 268, 410	None	8, 25, 81, 410	8, 22, 25, 34, 81, 121, 196, 268, 297, 408, 410	**8, 22, 25, 81, 268, 410**
Carnivora S	77, 108, 113, 139, 147, 194, 227, 239, 243, 244, 261, 294, 336, 344, 360, 461, 470, 477, 478, 556, 575, 578, 605, 607, 611, 630, 642, 645, 648, 663, 699, 701, 741, 752, 763, 776, 819, 837, 842, 892, 898, 1050, 1078, 1161, 1217,	77, 147, 227, 479, 609, 743, 894, 1080	None	147, 227, 244, 344, 360, 440, 462, 479, 480, 609, 613, 743, 1052, 1080, 1219	147, 227, 479, 609,	**147, 227, 462,** **479, 609**
Carnivoral E	None	None	None	None	None	-
Carnivora M	None	None	None	None	None	-
Carnivora N	384	None	None	384	None	-
Human S	2, 5, 12, 49, 75, 77, 78, 138, 139, 144, 147, 238, 243, 310, 343, 349, 352, 359, 383, 424, 435, 441, 462, 471, 479, 486, 500, 576, 599, 604, 607, 608, 612, 622, 651, 664, 665, 742, 764, 777, 793, 855, 859, 860, 862, 1000, 1131, 1147, 1162, 1168, 1182, 1207, 1222, 1246	None	None	12, 138, 311, 608, 609, 1148, 1163, 1208	138	**138**
Human E	5, 6, 23, 29	None	None	None	None	-
Human M	5, 11, 27, 38, 68, 73, 81, 86, 91, 99, 113, 119, 154, 210	None	None	11	None	-
Human N	None	None	None	None	None	-

* Note: positively selected codons: only the codons identified using at least three of the five methods were considered to be positively selected codons.“-” indicates that no positive selection site was detected in this gene.

**Table 3:** Selective pressure analyses of the S, E, M, and N genes from MERS-CoV.

Gene		Positive Selection Pressure Sites Identified Using Different Methods				
	M8 vs. M8a	FEL	SLAC	FUBAR	MEME	PSC ^a^
Bat S	None	3, 7, 25, 225, 328, 625, 731, 777	None	219	3, 7, 25, 145, 199, 222, 225, 232, 235, 239, 591, 687, 713, 731, 772, 777, 794, 795, 908, 966, 1277, 1293	-
Bat E	None	None	None	None	None	-
Bat M	None	None	None	None	96	-
Bat N	None	200, 328, 378, 389, 394, 402, 403, 424, 435	None	200, 328, 389, 398, 424	111, 200, 210, 328, 367, 378, 389, 402, 403, 406, 424, 431	**200, 328, 389, 424**
Camel S	26, 459, 465, 612, 723, 1193, 1224	26, 28, 424, 459, 723, 1224	26	26, 28, 158, 390, 424, 710, 723, 1193, 1224	26, 28, 424, 459, 723, 1224	**26, 28, 424, 459, 723, 1224**
Camel E	None	None	None	None	None	-
Camel M	None	69, 111, 155	None	8, 69, 82	69	**69**
Camel N	None	3, 198	None	3, 198	3, 198	**3, 198**
Human S	None	424	None	26, 91, 95, 301, 424, 507, 509, 534, 914, 1158	1020	-
Human E	None	None	None	None	None	-
Human M	None	None	None	15, 20, 69	None	-
Human N	None	None	None	8, 126, 300	None	-

Note: **PSC ^a^**: positively selected codons: only the codons identified using at least three of the five methods were considered to be positively selected codons.“-” indicates that no positive selection site was detected in this gene.

## Data Availability

The datasets generated and/or analyzed during the current study are available from the corresponding author upon reasonable request.

## Supplementary Materials

The following supporting information can be downloaded at: https://www.mdpi.com/article/10.3390/v17081114/s1, Table S1: GenBank accession numbers of public sequences used in this study; Table S2: The primers used in this study; Figure S1: Haplotype network of the S gene from SARS-CoV and SARSr-CoV genomes. (Year); Figure S2: Haplotype network of the S gene from MERS-CoV and MERSr-CoV genomes. (Year).
